# Laparoscopic Esophagogastric Anastomosis With Stapled Pseudo-Fornix for Reflux Esophagitis Prevention After Proximal Gastrectomy

**DOI:** 10.7759/cureus.25561

**Published:** 2022-06-01

**Authors:** Yusuke Fujii, Takashi Yasuda, Tatsuya Inoue

**Affiliations:** 1 Gastroenterological Surgery, Hyogo Prefectural Harima-Himeji General Medical Center, Himeji, JPN; 2 Course of Advanced Clinical Research of Cancer, Juntendo University Graduate School of Medicine, Tokyo, JPN

**Keywords:** pseudo-fornix, reflux esophagitis, esophagogastric anastomosis, gastric cancer, laparoscopic proximal gastrectomy

## Abstract

Laparoscopic esophagogastric anastomosis is not commonly performed after proximal gastrectomy (PG) because of its technical complexity and the lack of a gold standard for reconstruction. We describe a simple and convenient technique of laparoscopic esophagogastrostomy with stapled pseudo-fornix for reflux esophagitis (RE) prevention after PG.

Laparoscopic PG (LPG) was performed in four patients with gastric cancer in the upper third of the stomach, and the remnant stomach was prepared for reconstruction. After making a small hole on the anterior wall of the remnant stomach 45 mm distal to the proximal stump and on the dorsal side of the esophageal stump, a 45 mm no-knife linear stapler was applied. To create a "pseudo-fornix," a common lumen was made by cutting the center of the four staple rows at a length of 15 mm. The entry hole was closed using the laparoscopic hand-sewn suturing technique.

The mean operation time was 240 min, with an estimated blood loss of <10 ml. No intraoperative complications or conversion to open surgery were observed. One patient developed stenosis of the esophagogastrostomy successfully treated by endoscopic balloon dilatation. Endoscopic surveillance three months after surgery revealed no incidence of RE in any of the patients.

Laparoscopic esophagogastric anastomosis with stapled pseudo-fornix is convenient and beneficial in preventing RE after PG and should be considered the treatment of choice for reconstruction after LPG in selected patients with proximal gastric cancer.

## Introduction

Gastric cancer is one of the most common cancers worldwide. A large proportion of gastric cancers are detected at an early stage in Japan, and the incidence of early gastric cancer in the upper third of the stomach continues to increase despite the decrease in the total number of gastric cancers reported. Although total gastrectomy (TG) remains the principal surgical procedure for proximal gastric cancer, proximal gastrectomy (PG) can be used for selected patients as less invasive or function-preserving surgery.

PG has potential advantages over TG, such as preserving remnant gastric capacity and entailing fewer hormonal and nutritional deficiencies [1-3]. Esophagoenteric anastomosis following PG includes esophagogastrostomy and jejunal interposition or double-tract reconstruction. Esophagogastrostomy is considered to be a simple and more physiological reconstruction, requiring a shorter operation time than other reconstruction options [2-6]. However, a higher incidence of postoperative reflux esophagitis (RE) due to gastroesophageal regurgitation has been reported after esophagogastrostomy [2-7].

There are several techniques applicable for the prevention of gastroesophageal reflux when performing an esophagogastric anastomosis using either open surgery or a laparoscopic approach [4-7]. Meanwhile, laparoscopic esophagogastrostomy is not yet widely performed following laparoscopic PG (LPG), probably due to its technical complexity and the lack of a gold standard for reconstruction.

This report describes a simple and convenient technique of laparoscopic esophagogastrostomy with stapled pseudo-fornix for RE prevention after LPG using a no-knife linear stapler and the hand-sewn suturing technique, which is a procedural modification of esophagogastric tube reconstruction with stapled pseudo-fornix [8].

## Technical report

Patients and methods

Four patients with gastric cancer in the upper third of the stomach underwent LPG followed by laparoscopic esophagogastric anastomosis with stapled pseudo-fornix. The preoperative assessment of gastric cancer was conducted using endoscopy, endoscopic ultrasonography, upper gastrointestinal series, and contrast-enhanced computed tomography scans. The pathological classification of gastric cancer and the extent of lymph node dissection were based on the third English edition of the Japanese Classification of Gastric Carcinoma [9] and the Japanese Gastric Cancer Treatment Guidelines 2018 (fifth edition) [10]. All tumors included in this study were histologically verified gastric adenocarcinoma in the upper third of the stomach.

The postoperative complications were recorded and classified according to the Clavien-Dindo classification [11]. Postoperative endoscopic assessment of the esophagus and remnant stomach, including the esophagogastric anastomosis, was performed three months after surgery, and RE due to gastroesophageal regurgitation was examined and classified using the Los Angeles classification [12].

Surgical techniques

**Figure 1:**
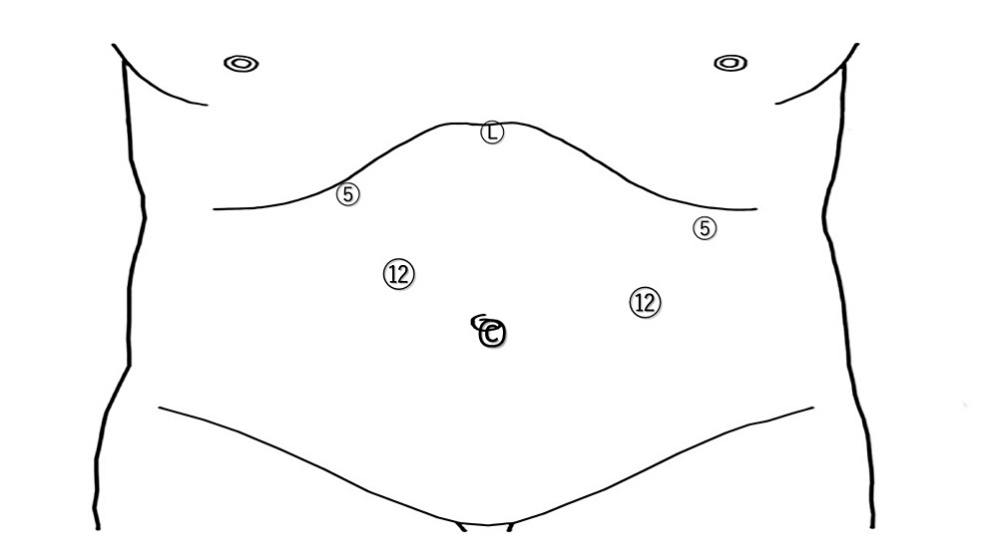
Port sites for laparoscopic proximal gastrectomy C: the camera port site; L: the Nathanson liver retractor site; 5: 5-mm port sites; 12: 12-mm port sites

The patient was placed in a supine position with the legs apart under general anesthesia. After creating a pneumoperitoneum at the umbilicus using an open method, a flexible laparoscope was introduced through the umbilical trocar. Four additional operating trocars were placed in the upper abdomen (Figure 1). The lateral segment of the liver was retracted using the Nathanson liver retractor prior to gastrectomy.

On the greater curvature, the left gastroepiploic artery and short gastric arteries were divided and the right gastroepiploic artery was preserved, i.e., the No. 4sb and 4sa lymph nodes were dissected [9]. Next, the suprapancreatic lymph nodes along the left gastric artery (No. 7), common hepatic artery (No. 8a), celiac artery (No. 9), and proximal splenic artery (No. 11p) were dissected [9] and the root of the left gastric artery was divided. The esophagus was transected horizontally using an endoscopic linear stapler after complete exposure of the abdominal esophagus by dividing the vagal trunks.

Then, the right pericardial (No. 1), left pericardial (No. 2), and lesser curvature (No. 3a) were completely dissected. When necessary, peri-esophageal and lower mediastinal nodes (No. 19, 20, 110, 111, and 112) and along the distal splenic artery (No. 11d) were also dissected in advanced cases [9-10].

**Figure 2:**
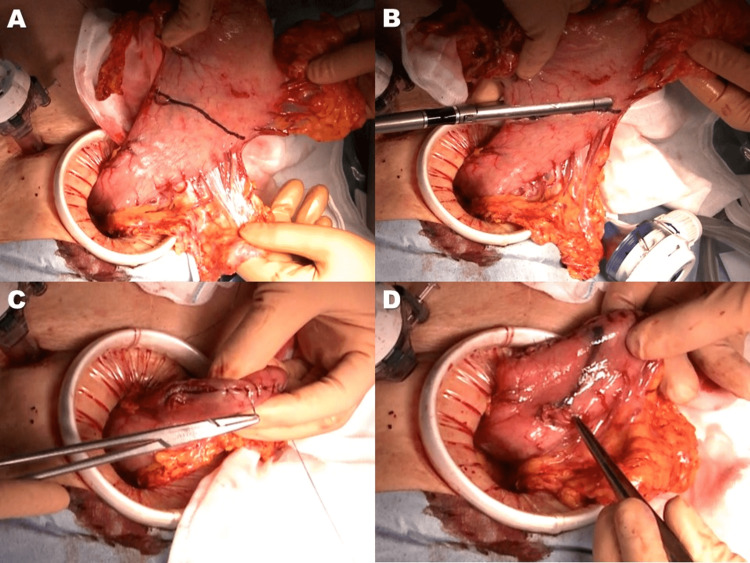
Completion of proximal gastrectomy and preparation of remnant stomach for reconstruction A) The transection line for the stomach was marked with Gentian Violet ink B) The stomach was transected using a linear stapler C) Seromuscular sutures along the stapled line of the remnant stomach D) A small hole was made on the anterior wall of the remnant stomach 45 mm distal to the proximal stump

The umbilical port incision was extended by approximately 5 cm in a craniocaudal direction to pull out the stomach. The planned transection line for the stomach was marked with Gentian Violet ink to secure the distal resection margin for the tumor and to achieve an adequate remnant stomach size (Figure 2A), and the stomach was transected using an endoscopic linear stapler outside the abdominal cavity (Figure 2B). Seromuscular sutures were added along the stapled line of the remnant stomach (Figure 2C). A small hole was then made on the anterior wall of the remnant stomach 45 mm distal to the proximal stump (Figure 2D).

A small laparotomy is required for specimen removal. The small laparotomy is not performed under the xiphoid process but at the umbilicus, considering the effect on postoperative respiratory function. The preparation of the remnant stomach for reconstruction and the anastomosis can be performed intracorporeally, but if a small laparotomy is required anyway, it is better to do what can be done outside the body to shorten the operation time. The preparation of the remnant stomach can be performed at the umbilical wound site, but anastomosis is not possible, so the anastomosis is performed intracorporeally.

**Figure 3:**
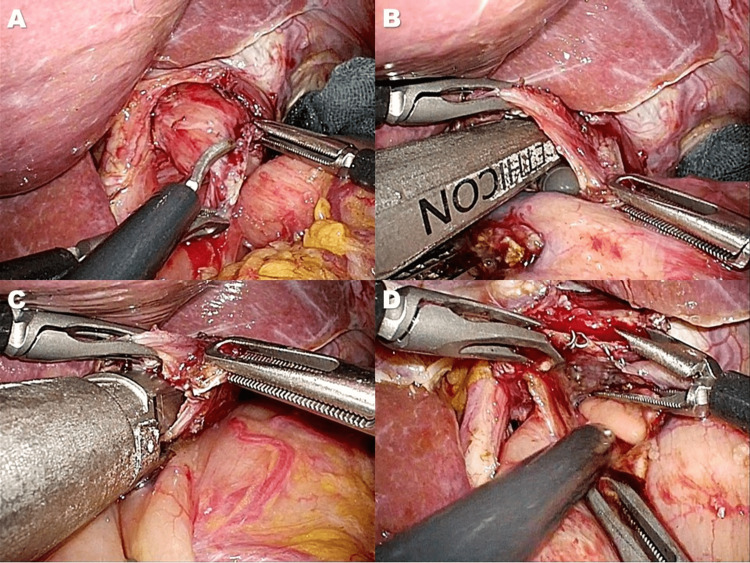
A connection between the esophagus and the remnant stomach was established using the linear stapler A) A small hole was made on the dorsal side of the esophageal stump B) A no-knife endoscopic linear stapler was applied on the anterior wall of the remnant stomach and the dorsal side of the esophagus under the guidance of a nasogastric tube C, D) A connection between the esophagus and the remnant stomach using the stapler (C) without constructing a common lumen (D)

**Figure 4:**
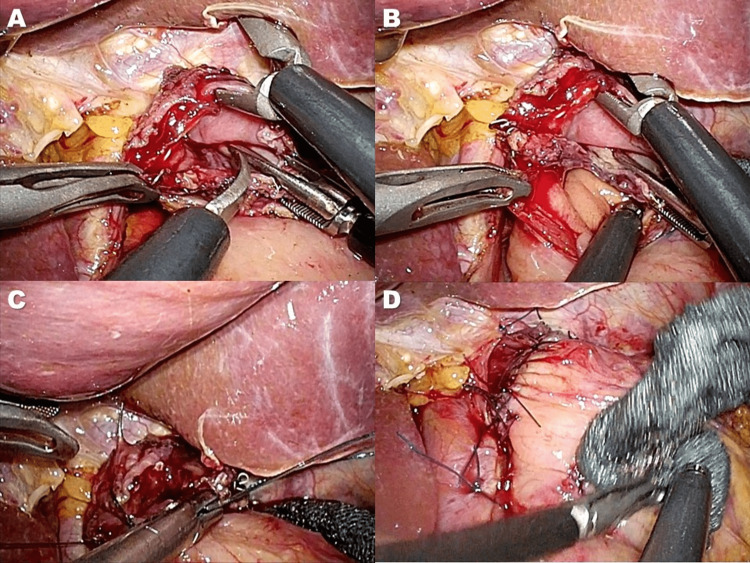
Creation of V-shaped anastomosis and completion of reconstruction A) A common lumen was made by cutting the center of the 4 staple rows at a length of 15 mm to create a pseudo-fornix B) A small V-shaped anastomosis was created C) The entry hole was closed using the laparoscopic hand-sewn suturing technique D) The abdominal esophagus near the anastomotic site was sutured to the crus of the diaphragm

**Figure 5:**
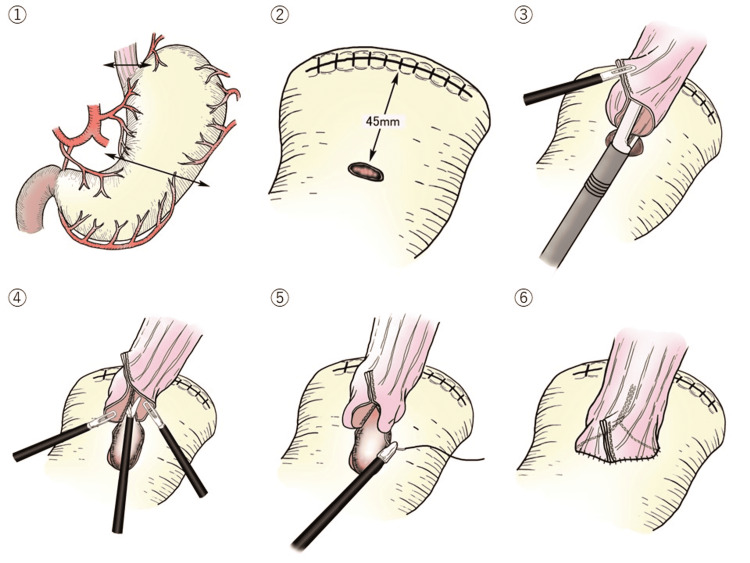
The outline of this procedure The outline of the laparoscopic esophagogastric anastomosis with stapled pseudo-fornix following proximal gastrectomy ① The stomach was transected using a linear stapler outside the abdominal cavity. ② A small hole was then made on the anterior wall of the remnant stomach 45 mm distal to the proximal stump. ③ The stapler established a connection between the esophagus and the remnant stomach without creating a common lumen. ④ A common lumen was made by cutting the center of the 4 staple rows at a length of 15 mm to create a pseudo-fornix. ⑤ The entry hole was closed using the laparoscopic hand-sewn suturing technique. ⑥ <Completion of anastomosis> A small V-shaped anastomosis was created.

Subsequently, a small hole was made on the dorsal side of the esophageal stump (Figure 3A). An ETS-Flex 45 no-knife endoscopic articulating linear stapler (Ethicon Endo-Surgery, Tokyo, Japan) was applied to the anterior wall of the remnant stomach and the dorsal side of the esophagus under the guidance of a nasogastric tube (Figure 3B). The stapler created a connection between the esophagus and the remnant stomach (Figure 3C) without constructing a common lumen (Figure 3D). To create a ‘‘pseudo-fornix,’’ a common lumen was made by cutting the center of the four staple rows at a length of 15 mm (Figure 4A), forming a small V-shaped anastomosis (Figure 4B). The entry hole was closed using the laparoscopic hand-sewn suturing technique (Figure 4C). Finally, the abdominal esophagus near the anastomotic site was sutured to the diaphragmatic crus to prevent hiatal hernia development (Figure 4D). Figure 5 outlines this procedure.

Results

Four patients underwent LPG followed by laparoscopic esophagogastric anastomosis with pseudo-fornix using a no-knife linear stapler. Two patients had early-stage gastric cancer and two had advanced cancer. Three patients had associated severe comorbid disorders, such as diabetes mellitus, obesity, chronic heart failure, chronic renal failure, or chronic obstructive pulmonary disease. Curative resection was achieved in three patients. The remaining patient underwent a palliative LPG without regional lymph node dissection. This patient had advanced gastric cancer with peritoneal dissemination and underwent LPG for the management of bleeding and narrowing of the esophagogastric junction due to cancer.

**Figure 6:**
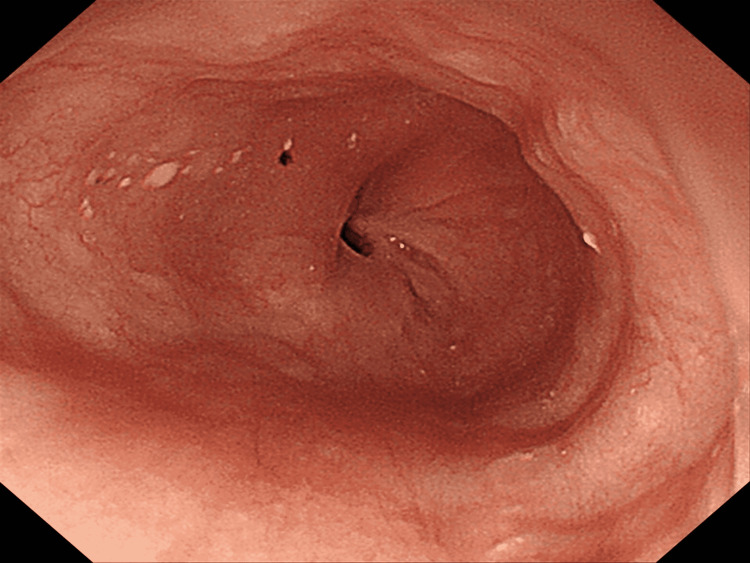
Endoscopic finding three months after esophagogastrostomy Endoscopic finding three months after esophagogastrostomy revealed no reflux esophagitis

**Figure 7:**
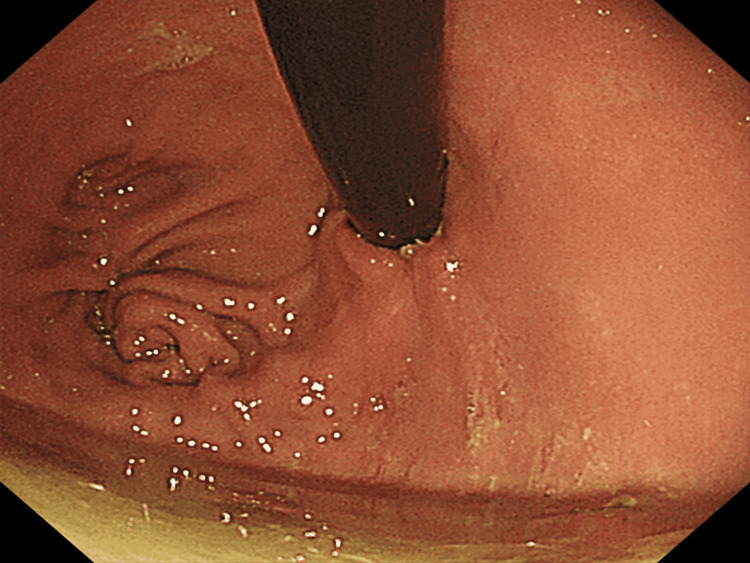
Newly formed pseudo-fornix Endoscopic finding three months after esophagogastrostomy demonstrated the newly formed pseudo-fornix to be well-maintained

The mean operation time was 240 (226-283) min and the estimated blood loss was < 10 ml. There were no intraoperative complications, and there was no need or conversion to open surgery. Furthermore, there were no severe postoperative complications or operative mortality after surgery, except for stenosis of the esophagogastrostomy (Clavien-Dindo classification grade IIIa) in one patient. In this patient, the anastomotic stenosis was successfully treated by endoscopic balloon dilatation. The median postoperative hospital stay was 14 (11-32) days. All patients were routinely administered proton pump inhibitors postoperatively, and none of them had reflux symptoms. Endoscopic examination three months postoperatively revealed no incidence of RE in any of the patients (Figure 6), and the newly formed pseudo-fornix was well-maintained (Figure 7).

With a median 14 months follow-up period of 14 months, no recurrence was observed in three patients who underwent curative resection. The patient who underwent palliative gastric resection received optimum supportive care without chemotherapy and was doing well six months after surgery.

## Discussion

The choices for gastric cancer surgical methods primarily depend on tumor size and location. TG is the standard surgical option for either early or advanced gastric cancer in the upper third of the stomach. PG is a possible treatment of choice for early-stage gastric cancer in open surgery [13-14]. The primary goal of the operation is to completely remove the tumor with negative surgical margins and adequate lymphadenectomy, i.e., R0 resection [10,13-14]. The survival outcome of PG for proximal gastric cancer is equal to that of TG, and besides, PG has some advantages over TG such as preserving remnant stomach and fewer hormonal deficiencies [13-14]. In addition, PG with esophagogastric reconstruction provides several major benefits, including a shorter operative time, less postoperative weight loss, and fewer nutritional deficiencies, than TG [3]. Therefore, it is beneficial to establish a simple and useful esophagogastric anastomosis following PG.

Recently, laparoscopic gastrectomy has been widely accepted for the surgical treatment of early-stage gastric cancer worldwide. However, LPG is not yet commonly performed, probably due to the lack of a standard reconstruction technique that is convenient and efficient against postoperative gastroesophageal reflux. A jejunal interposition was initially proposed to achieve a safe anastomosis with anti-reflux [15], but it would be a complicated maneuver. Linear stapled esophagogastrostomy was developed as a simple and convenient procedure [16-18]. Fundoplication with anastomotic wrapping or forming a flap valve was employed as an anti-reflux procedure [7,16-18]. The double-flap technique would be a more physiological reconstruction and useful for reducing postoperative RE; however, it is also a complicated and time-consuming procedure for laparoscopic surgery [7]. Recently, an anastomosis between the posterior wall of the esophagus and the anterior wall of the remnant stomach following PG was demonstrated, creating a new notch at the dorsal side of the anastomosis to prevent postoperative RE [7,16-18].

Okabe et al. and Hosogi et al. recently developed an innovative esophagogastric anastomosis using a no-knife linear stapler in patients undergoing LPG [4,8]. In Okabe’s anastomotic procedure, a 45-mm-long stapler was used and the rows of staples were left uncut for the ‘‘pseudo-fornix’’ formation, and then both the posterior and anterior walls were closed entirely using the hand-sewn suturing technique. In Hosogi’s anastomotic procedure, a 30-mm-long stapler was used for the “pseudo-fornix” formation, and the anterior wall was closed by hand suturing, where the remnant stomach was prepared for a narrow gastric conduit. Incorporating the benefits of these procedures, we developed a reconstruction procedure that can provide technical simplicity and a large residual stomach capacity in terms of a function-preserving gastrectomy. Our procedure requires a relatively small amount of hand-sewing closure, which may facilitate the flexibility and simplicity of esophagogastric anastomosis while shortening the operation time.

This study has some limitations. RE evaluation was conducted using limited information from endoscopic findings and not through a validated questionnaire. The small sample size and the lack of generalizability of the surgical approaches and/or procedures were also limitations of this study, as the bias of patients’ backgrounds could not be excluded.

## Conclusions

Laparoscopic esophagogastrostomy with stapled pseudo-fornix following LPG is a simple and feasible reconstruction procedure, in which the fixation of the esophagus to the anterior wall of the stomach with a no-knife linear stapler facilitates the easy creation of fundoplication to prevent postoperative RE. Although large prospective randomized controlled trials should be conducted to confirm our preliminary findings, we believed that our reconstruction procedure would be one of the beneficial options for selected patients with proximal gastric cancer.
